# The Book World of Medicine and Science

**Published:** 1893-10-28

**Authors:** 


### THE BOOK WORLD OF MEDICINE AND SCIENCE.

A Treatise on Public Health and its Applications in Different European Countries. By Albert Palmberg, M.D. Translated and edited by Arthur Newsholme, M.D., Medical Officer of Health for Brighton. pp. 540. Illustrated. (Swan Sonnenschein and Co. New York: Macmillan and Co., 1893.)

English readers interested in public health owe a very distinct debt of gratitude to Dr. Newsholme for presenting to them in their own tongue this valuable contribution to the literature of the subject in a volume unequalled in interest since the publication of Sir John Simon's "English Sanitary Institutions." In the earlier volume the growth and progress of public health in England has been traced from the earliest times, and now in this volume we are taken further afield and are presented with a comparative view of English and Continental sanitary organisation as it exists at the present day. No method is so helpful in education as the comparative method, and it is a distinct gain for the English student of public health, who is somewhat apt to tacitly accept the home system as perfect, to see how in many matters, notably as to food inspection in France and other countries, and as to compulsory revaccination in Germany, there is much worthy of imitation in the practice of other nations.

The sections of the work deal with England, in chapters revised and brought up to date by the translator, Scotland, Belgium, France, Germany, Austria, Sweden, and Finland, and in each country the sanitary organisation of the chief town receives special notice. The chapters on England and London are, on the whole, carefully edited, and would give a foreigner a fairly accurate notion of our sanitary organisation; in a volume dated 1893, however, it should have been possible to include notice of the later cholera order of 1890, which supersedes the order of 1883 quoted in the text (p. 35); and of later rag orders than that of September, 1890. On page 115 the standards of the Society of Public Analysts appear to be given too high, and on page 159 transposition of the titles to the woodcuts makes the text somewhat confusing. But it is only fair to say that these and one or two other evidences of haste in the preparation of the article are fully atoned for by the general excellence of its information.

The sections dealing with the collection and disposal of sewage in the various countries dealt with are full of interest, and much is to be learnt from their perusal. The abundance of illustrations renders the text very clear, and in this way curious habits, such as the continued use in France of the water closet à la Turque, are forcibly delineated The inclusion of several figures from the annotated model bye-laws of Messrs. Knight makes the section on English sanitation more complete.

*Vaccination* must, in England, be performed before a child reaches the age of three months; in Germany children under three months must not be vaccinated unless there is an epidemic of small-pox, but every child must be vaccinated in the year which follows that of its birth, and all pupils of public or private schools, Sunday schools and evening schools excepted, must be revaccinated in their twelfth year, unless they have had small-pox or have been successfully vaccinated within five years. In Austria, vaccination is not generally compulsory, except during an epidemic of small-pox, but the State exerts itself to induce the people to adopt it; all who

**THE BOOK WORLD OF MEDICINE AND SCIENCE**—*continued.*

desire admission into a public school, or wish to obtain a scholarship, must produce a certificate of vaccination. Very similar conditions obtain in France. In Sweden all children have to be vaccinated before the age of two years, and no one can be admitted into a school or public establishment unless it is certain that he has either had small-pox or been successfully vaccinated, or that vaccination performed five years before produced no results; and if small-pox has already broken out, all unvaccinated persons must be vaccinated without delay, at any season and without distinction of age.

In Finland children must also be vaccinated before they are two years old, and if small-pox breaks out in any place, al unvaccinated persons must be vaccinated without delay. In Sweden and in Finland other persons than doctors holding diplomas are permitted to perform the operation. The volume does not contain any controversial consideration of the vaccination question.

*Notification* of infectious diseases is in England already in force amongst five-sixths of the population, although the Act of 1889 is "adoptive" and not compulsory. In Paris no compulsory notification of infectious diseases is enforced except for lodging-houses. In Germany notification is compulsory upon every father of a family, occupier, hotel-keeper, doctor, or clergyman, and is made to the police, who obtain medical assistance; and many of the preventive measures during epidemics are also under police control. In Vienna the notifiable diseases include, in addition to those scheduled in the English Act, dysentery, purulent ophthalmia, hydrophobia, and trichinosis, and notification is incumbent upon the medical attendant. There are no separate infectious disease hospitals in Vienna, but there are special pavilions for such diseases in connection with the general hospitals. In Sweden, upon the outbreak of an epidemic, the medical services must at once be organised. Every doctor attending a case of infectious disease must notify, and persons suffering from epidemic or infectious disease cannot refuse to be removed to the special hospital unless life would be endangered, or they can be nursed in a place properly isolated at their own expense. In Finland the owner of a house and the father of a family must immediately inform the Health Commission of any case of infectious disease.

*Sanitary Regulations Respecting Provisions.*—The arrangements existing in Paris for the inspection of meat and the control of abattoirs are singularly complete, and will repay careful study. It is much to be desired that all large centres of population in England should provide abattoirs in which all slaughtering must be performed under efficient inspection and control, as by this means alone can a proper supervision of the meat supply be exercised.

It would be easy, did space permit, to quote many other interesting details of sanitary administration and appliances in the various countries in regard to water supplies, ventilation, hospital construction, building regulations, factories, housing of the working classes, disinfection, sewerage and drainage, and quarantine, but sufficient has been said to show the scope and value of the book, which deserves careful perusal. The work is excellently printed, bound, and illustrated.

THE BOOK WORLD OF MEDICINE AND SCIENCE—(*continued*).

### FIRST IMPRESSIONS OF BOOKS RECEIVED.

[It is requested that all books intended to be noticed in this column may be sent to the Editor, at the Office, 428, Strand, W.C., not later than Tuesday evening in each week.]

Sciatic Neuritis; Its Pathology and Treatment. By Robert Simpson, L.R.C.P., L.R.C.S. (Bristol, John Wright and Co. 1893.)

This little brochure is published in a size and form which makes it tempting to the reader. Mr. Simpson has devoted special attention to this most troublesome malady, and the methods of treatment suggested, though making no claim to novelty, are useful and practical. As Mr. Simpson observes, the general adoption of massage as a therapeutic agent is still a matter of the future, but a wider experience of its utility will probably occasion its more general application. Mr. Simpson treats the subjects under three heads, which include the maintenance of nutrition in each of the great nerve trunks of the body; the nature of the pathological changes occurring in neuritis; and the nature and action of various theriapeutic agents. The book contains 46 pages, and the published price is one shilling.

The Value of Hypnotism. By Thos. Crisfield, Masseur, &c., London. Price one shilling.

Mr. Thos. Crisfield, Masseur, &c., who carefully appends his full address to his preface, writes a pamphlet "to re-assure timid people who have a sort of general impression that the entire subject (of hypnotism) is in some way 'uncanny' and had better be left religiously alone." We "have a sort of general impression" that Mr. Crisfield wishes to advertise himself. His cases are the old familiar ones of alcoholism cured by suggestion, yet he assures that his dipomaniacs were by no means weak-minded persons yielding temporarily to the force of a stronger will. If they were strong-minded how did they become drunkards? He assures us, however, that "hypnotism has proved of the greatest value in cases of chorea, St. Vitus's dance, chronic movements, asthma, ovarian pains, neurasthemia, spinal irritation, vertigo, spontaneous somnambulism, uneasy dreams, moral depravity, palpitation, nervous disorders, of sight, tinnitus aurium, loss of apetite, vomiting, chlorosis, anæmia, &c." This is a large order.

Letts' Calendars and Diaries. (Charles Letts and Co., London.)

Messrs. Letts publish a great variety of diaries, and the name of this firm has become a household word in this connection. From the "Improved Diary" of quarto size, which can be had for sixpence, to the dainty "Waistcoat-pocket" dairy, elegantly bound in morocco, a high level of merit is maintained. Taken as a whole these diaries meet every phase of human requirement, but we personally should give the preference to the "Improved Pocket Diary and Cash Book," which for people of business habits is without a peer.

A New Visiting List.

Wrights' "Improved Physicians', Surgeons', and Consultants' Visiting List," compiled by Robert Simpson, L.R.C.P., and published by J. Wright and Co., of Bristol, presents several new and useful features. It is handy and admirably arranged. In addition to the visiting list proper, it contains a "Consultant's Record," space for obstetric engagements, nurses' addresses, and about a dozen tables, including a list of principal drugs and their doses. Taken altogether, we consider this the best Practitioner's Visiting List ever published.

